# Cutaneous Collagenous Vasculopathy: A Rare Entity Treated With Pulsed Dye Laser

**DOI:** 10.7759/cureus.58391

**Published:** 2024-04-16

**Authors:** Ganesh B Maniam, Nneka Comfere, Giang H Nguyen

**Affiliations:** 1 Dermatology, Mayo Clinic, Rochester, USA; 2 Laboratory Medicine and Pathology, Mayo Clinic, Rochester, USA

**Keywords:** pulsed dye laser, vasculopathy, cutaneous collagenous vasculopathy, dermatopathology, dermatology

## Abstract

Cutaneous collagenous vasculopathy (CCV) is a rare idiopathic dermal microangiopathy. Clinically, it presents as diffuse cutaneous telangiectasias that are indistinguishable from other benign vascular entities, thereby posing a diagnostic challenge. We present a case of CCV successfully treated with pulsed dye laser (PDL). A 27-year-old male presented with generalized erythematous macules, diagnosed as CCV via histopathology. After a successful test spot, PDL treatment resulted in significant improvement. The pathogenesis of CCV involves altered dermal microvasculature and veil cell activation. Epidemiologically, it primarily affects Caucasians, most often in the middle-aged adult population. A negative family history of similar lesions can help narrow down the differential diagnosis. Diagnosis requires biopsy, with histopathological examination demonstrating vessel ectasia and collagenous vessel wall thickening. Given its rarity, CCV presents diagnostic and management challenges though PDL emerges as a promising treatment modality for this condition.

## Introduction

Cutaneous collagenous vasculopathy (CCV) is a rare type of idiopathic dermal microangiopathy that was first reported in 2000 [1]. This presents as diffuse cutaneous telangiectasias which are clinically indistinguishable from other vascular disorders and thus present a diagnostic challenge [2-4]. It presents as asymptomatic blanchable macules and telangiectasias, most often starting on the lower extremities before spreading upward [2]. This condition has been reported in the middle-aged population, with some sources suggesting a female predominance [2]. It has also been reported with underlying cardiovascular disease and diabetes mellitus [2]. The diagnosis of CCV requires histopathological examination demonstrating not only vessel ectasia but also a component of collagenous vessel wall thickening due to deposition of hyaline material [1-4]. Acquired telangiectasias are often not biopsied, and thus, CCV may be underdiagnosed and underreported in the literature [2-3]. This case presents a young male with CCV that was successfully treated with pulsed dye laser (PDL).

## Case presentation

**Figure 1:**
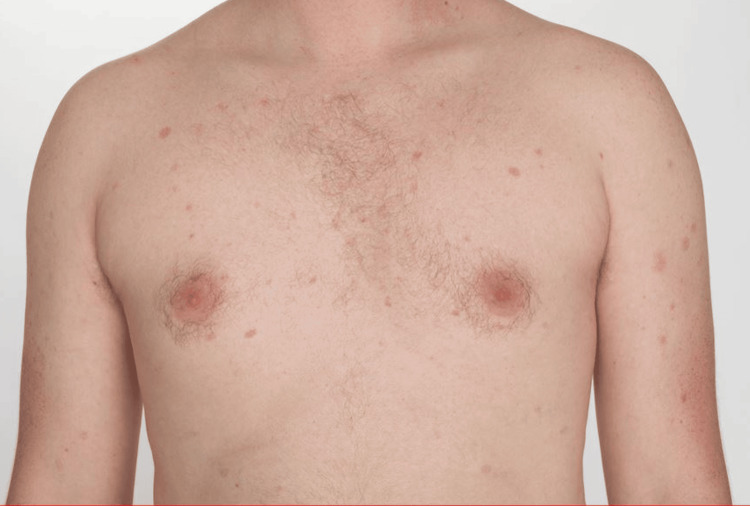
Photograph of blanchable erythematous macules seen on the chest and upper extremities

A 27-year-old male presented with generalized erythematous fully blanchable macules, some of which were annular in morphology (Figure 1).

**Figure 2:**
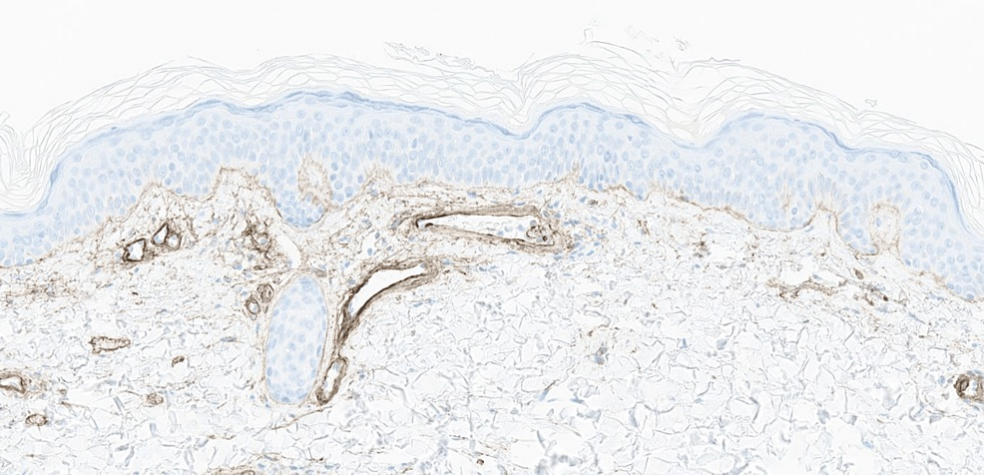
Type IV collagen staining highlights a slight increase in superficial dermal vessel wall thickening (collagen IV, 160x)

**Figure 3:**
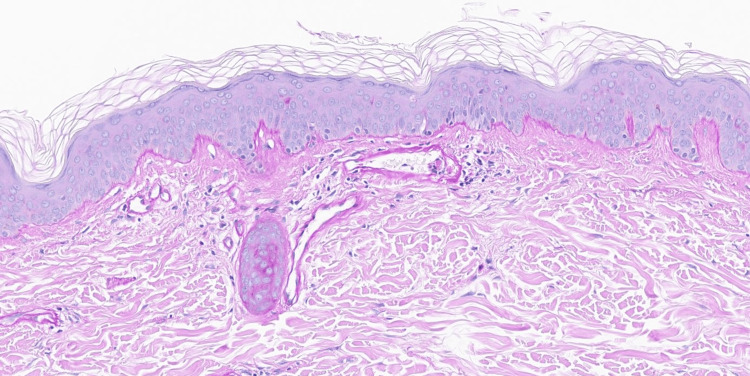
Periodic acid-Schiff (PAS) staining highlights a slight increase in superficial dermal vessel wall thickening (PAS, 160x)

These lesions reportedly started approximately two years ago, initially on the thighs before spreading to the trunk and arms. These lesions were reportedly asymptomatic but cosmetically bothersome. Laboratory studies for antineutrophil cytoplasmic antibodies, antinuclear antibodies, complement, and cryoglobulins were unremarkable. A punch biopsy was performed. Histopathological examination demonstrated vascular ectasia, while periodic acid-Schiff (PAS) and type IV collagen staining highlighted a slight increase in superficial dermal vessel wall thickening (Figures 2-3).

**Figure 4:**
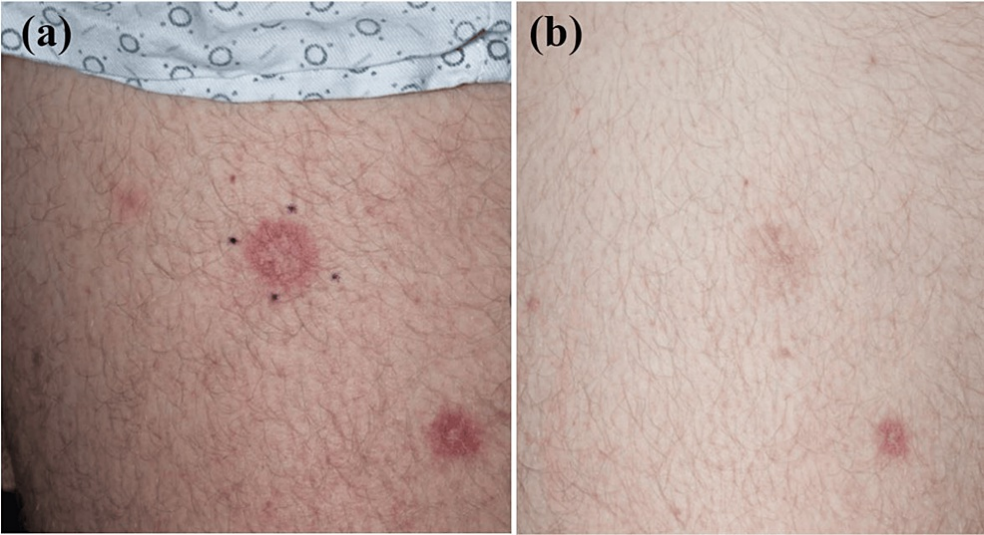
A lesion on the left anterior thigh is seen before (4A) and after (4B) one treatment with pulsed dye laser

After test spots demonstrated significant improvement (Figures 4A-4B), targeted laser treatment was subsequently performed to remaining lesions via PDL 585 nanometers at a fluence of 8 Joules per centimeter squared, pulse duration of 2 milliseconds, and spot size of 7 millimeters. The patient tolerated the procedure well without complications.

## Discussion

The composition and cell morphology of dermal microvasculature are known to be altered in inflammatory skin diseases, cutaneous neoplasms, and chronological aging. Veil cells, also known as periadventitial cells, are immature dendritic cells that surround these microvessels and are known to increase in both size and number in certain conditions [3,5]. While the pathogenesis of CCV has not been fully elucidated, it has been proposed that these veil cells are activated in response to endothelial cell injury. This may trigger abnormal and disorganized collagen deposition in the walls of dermal microvasculature via reparative fibrosis [1,3]. 

The epidemiological characteristics of CCV are limited by the sparsity of cases, as there are fewer than 100 cases reported in the literature. However, acquired telangiectasias are often not biopsied, and thus, this may lead to underreporting [2-3]. This condition is primarily seen in Caucasians, and middle-aged to older adults seem to be almost exclusively affected by this condition [1]. Thus, our case is atypical in that a young male developed CCV. There have also been reported associations with diabetes mellitus, autoimmune conditions, and cardiovascular disease [1]. 

CCV typically begins as diffuse cutaneous telangiectasias on the lower extremities before spreading elsewhere, typically sparing the head and neck [2-4]. There is rarely variation in morphology among CCV reports, such as lesions with ecchymoses, petechiae, hyperpigmentation, and papules [3]. Clinically, the diagnosis is complicated as the presentation is indistinguishable from other vascular disorders such as generalized erythematous telangiectasias, hereditary hemorrhagic telangiectasia, and hereditary benign telangiectasia; however, it is worth noting that these other conditions are inherited in an autosomal dominant fashion; thus, a negative family history of similar lesions can be helpful in narrowing down the differential diagnosis [2-4]. Of note, CCV is not associated with systemic involvement and does not affect the hair, nails, or mucosal surfaces. 

The diagnosis of CCV requires a biopsy demonstrating dilation of superficial dermal vessels, including both capillaries and postcapillary venules, along with characteristic thickening of vessel walls due to type IV collagen deposition [1-4]. Within the vessel wall, there will be amorphous deposits of eosinophilic material which is highlighted by staining via PAS and type IV collagen, as well as iron colloidal stain and fibronectin [1-4]. This is a chronic and progressive disease but is asymptomatic with a benign course. However, the widespread distribution of these lesions may cause distress, and many patients desire treatment due to the cosmetic appearance [1-4]. Management options are limited, but prior reports have demonstrated efficacy with PDL as a treatment modality [3-4], and this was found to also be effective in this case.

## Conclusions

In conclusion, CCV represents a diagnostic enigma due to its rarity and clinical resemblance to other vascular disorders. Our case highlights the successful use of PDL in managing CCV-associated cutaneous manifestations, underscoring its potential as an effective therapeutic option. However, the limited understanding of disease pathogenesis and the lack of standardized treatment protocols both emphasize the need for further research on this entity. While this vasculopathy is a benign entity, patients may report cosmetic concerns due to its appearance. Thus, collaborative efforts and further research are essential to enhance our knowledge and improve clinical outcomes for affected individuals.
